# PFAAT version 2.0: A tool for editing, annotating, and analyzing multiple sequence alignments

**DOI:** 10.1186/1471-2105-8-381

**Published:** 2007-10-11

**Authors:** Daniel R Caffrey, Paul H Dana, Vidhya Mathur, Marco Ocano, Eun-Jong Hong, Yaoyu E Wang, Shyamal Somaroo, Brian E Caffrey, Shobha Potluri, Enoch S Huang

**Affiliations:** 1 Pfizer Global Research and Development, 620 Memorial Drive, Cambridge, MA 02139, USA; 2 Department of Electrical Engineering and Computer Science, Massachusetts Institute of Technology, MA 02139, USA; 3 Bioinformatics Program and Biomedical Engineering Department, Boston University, MA 02215, USA; 4 School of Theoretical Physics, Dublin Institute for Advanced Studies, 10 Burlington Road, Dublin 4, Ireland

## Abstract

**Background:**

By virtue of their shared ancestry, homologous sequences are similar in their structure and function. Consequently, multiple sequence alignments are routinely used to identify trends that relate to function. This type of analysis is particularly productive when it is combined with structural and phylogenetic analysis.

**Results:**

Here we describe the release of PFAAT version 2.0, a tool for editing, analyzing, and annotating multiple sequence alignments. Support for multiple annotations is a key component of this release as it provides a framework for most of the new functionalities. The sequence annotations are accessible from the alignment and tree, where they are typically used to label sequences or hyperlink them to related databases. Sequence annotations can be created manually or extracted automatically from UniProt entries. Once a multiple sequence alignment is populated with sequence annotations, sequences can be easily selected and sorted through a sophisticated search dialog. The selected sequences can be further analyzed using statistical methods that explicitly model relationships between the sequence annotations and residue properties. Residue annotations are accessible from the alignment viewer and are typically used to designate binding sites or properties for a particular residue.

Residue annotations are also searchable, and allow one to quickly select alignment columns for further sequence analysis, e.g. computing percent identities. Other features include: novel algorithms to compute sequence conservation, mapping conservation scores to a 3D structure in Jmol, displaying secondary structure elements, and sorting sequences by residue composition.

**Conclusion:**

PFAAT provides a framework whereby end-users can specify knowledge for a protein family in the form of annotation. The annotations can be combined with sophisticated analysis to test hypothesis that relate to sequence, structure and function.

## Background

Building a multiple sequence alignment (MSA) is a critical step towards understanding the function and evolution of a protein family. Subsequent analysis typically includes phylogenetics, homology modeling, structure prediction, and binding site prediction. There are several excellent software packages that align multiple sequences [1]. Alignment accuracy is usually dependent on the percent amino acid identity between sequences [2] and manual editing is often a necessary step. Alignment editing tools are available in PFAAT as well as several other applications [3-10]. Additionally, MSA viewers provide various tools for sequence and structural analysis [3,5-7,11-16]. More recently, it has been recognized that MSAs can be used to validate and propagate annotations to other sequences [17]. PFAAT specializes in the annotation and analysis of a MSA, and since the release of version 1.0 [18], we have continued to develop and add novel features to PFAAT. We describe some of the main features below.

## Implementation

PFAAT is written in Java and runs on several operating systems (Linux, Mac OS X, Solaris, and Windows). Users initially download and install the program from the home page using Java Web Start technology. Updated versions of the application are automatically downloaded on subsequent launches if the user is connected to the internet; otherwise the cached executable is used. Although PFAAT was not explicitly implemented for viewing nucleotide alignments, many of the generic features can also be applied to nucleotide sequences.

## Results and Discussion

**Figure 1:**
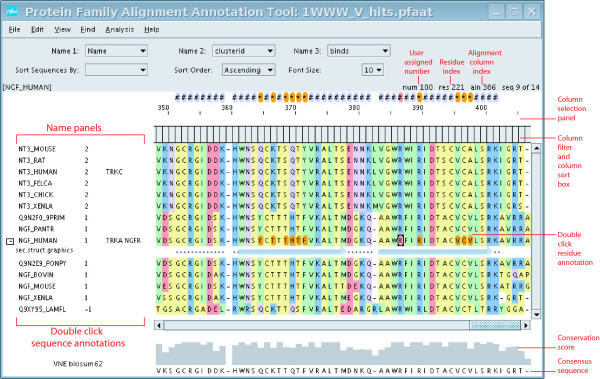
A screen shot of the alignment viewer. The red text highlights annotations along with various other features. The top row of drop-down menus was used to set the displayed sequence annotations in the name panels.

The alignment viewer is shown in Figure 1. The alignment can be edited in a number of ways. A gap can be inserted with the space bar or by dragging residues to the right while holding the *SHIFT *key down. A gap can be deleted with the *BACKSPACE *button or by dragging residues to the left while holding the *SHIFT *key down. Selecting multiple sequence names with the *CTRL *or *SHIFT *key allows one to edit a collection of sequences simultaneously. One can easily delete alignment columns that are composed entirely of gaps (*Edit -> Delete -> All Gap Columns*).

Double clicking on any of the three Name Panels will display the sequence annotations dialog box. Double clicking on a residue will display the residue annotation dialog box. The Tree viewer and structural viewer can be launched from the Analysis and File menus respectively. The tool bar (Figure 1) contains several drop-down menus that change the alignment view. The top row of drop-down menus changes the displayed annotation in Name Panels 1–3. The bottom row of drop-down menus sort sequences by annotation value and changes the font size.

### Sequence Annotations

**Figure 2:**
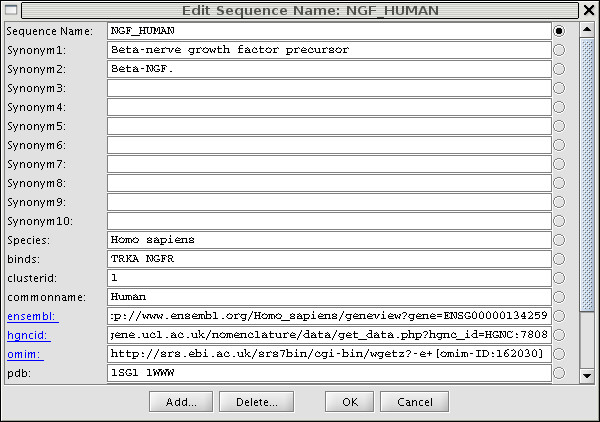
Sequence annotation dialog for NGF_HUMAN.

A sequence annotation provides a convenient way to assign a name and a value to one or more sequences in the alignment. Figure 2 shows all sequence annotations for a sequence after double clicking on Name Panel 1. The currently displayed annotation is indicated by the radio button and the text field allows editing of the annotation values. Sequence annotations include but are not limited to synonyms, species, and cluster IDs. The easiest way to create a new annotation name and value is to click on the *Add *button. We recommend adding a UniProt entry name, as it can be used later to automatically extract sequence annotations from UniProt [19]. PFAAT mines several UniProt fields which include synonyms, species names, PDB codes [20], and hyper-linked ENSEMBL [21] IDs. The user documentation on the PFAAT home page describes other ways to create sequence annotations easily.

When working with a large number of sequences, sequence annotations facilitate rapid sorting and triaging of sequences. For example, the *Find *menu allows one to find and select sequences that match one or more search terms (e.g. species *equals *Homo sapiens *AND *Pdb *is not empty*). The selected sequences can then be moved to the top using *View -> Sort Sequences by -> selection*.

### Residue Annotations

**Figure 3:**
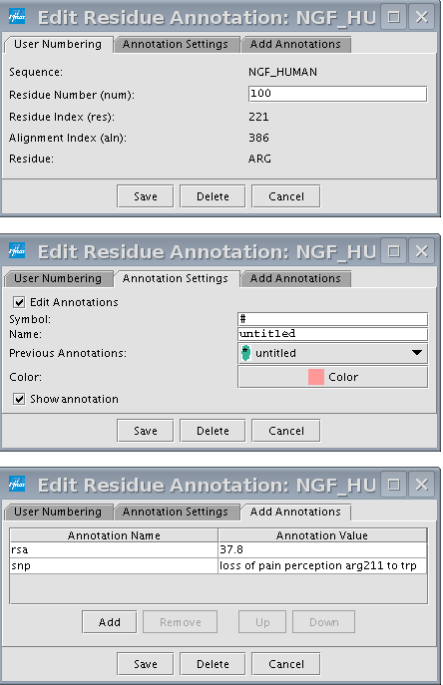
Residue annotation dialog for a residue in NGF_HUMAN. There is a screen shot for each tab.

Residue annotations are a useful way to flag residues of interest. They are typically used to specify binding sites, SNPs and post-translational modifications. Figure 3 shows the residue annotation dialog box that appears after one double clicks on a residue. The first tab displays various numbering for the residue and allows users to assign their own numbering. This field is particularly useful when the user omits domains or segments of sequence that will offset the default numbering. The second tab allows the user to specify how a residue annotation will appear. Each residue annotation must have a color and a symbol that is displayed above the alignment. There is also a check box that allows the user to show or hide the residue annotation. The third tab is where all annotation names and values are specified. After clicking on the *Save *button, the annotation will appear as a residue with a colored oval. The annotations can be viewed by mousing over the residue or double clicking the residue again.

Residue annotations provide a gateway for several types of subsequent analysis. For example, the *Find *menu allows one to quickly find and select residue annotations that match one or more search terms. The residue selection can be extended to the alignment column, and there is an option to hide all other columns. As a next step, one might apply one of the many features that can be applied to selected columns, including sorting by percent identity and most of the features in the *Analysis *menu.

### Phylogenetic Analysis

PFAAT reconstructs phylogenetic trees using an implementation of the neighbor joining algorithm [22]. An option to perform bootstrap analysis is also provided. Trees can be reconstructed using selected sequences or selected columns. PFAAT recognizes various tree formats (nh, nhx, nexus) and can display tree files generated by other software.

**Figure 4:**
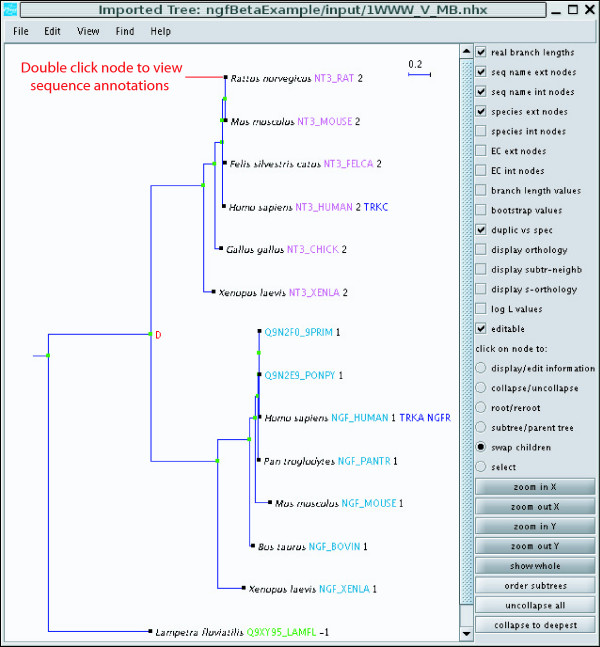
The tree viewer. Cluster IDs (black text) are assigned based on the gene duplication event and names are colored by cluster ID. The "binds" sequence annotation is displayed in blue text.

The tree viewer (Figure 4) borrows much of its code from ATV [23]. We have since added a number of new features that are tightly linked to the alignment viewer. Sequence annotations can be accessed by double clicking on the terminal nodes. The tree viewer has the same advanced searching capabilities as the alignment viewer. Sequence selection is synchronized between the tree viewer and the alignment viewer, allowing the user to quickly triage sequences based on evolutionary relationships. Another popular feature is the assignment of the cluster IDs in the tree viewer. Cluster IDs can be assigned based on gene duplication events or by dragging a vertical bar from left to right so it cuts the tree into clusters of a desired average size. As the cluster ID is a sequence annotation, it can also be used to sort sequences in the alignment viewer or to compute conservation scores for particular clusters.

### Structural Analysis

**Figure 5:**
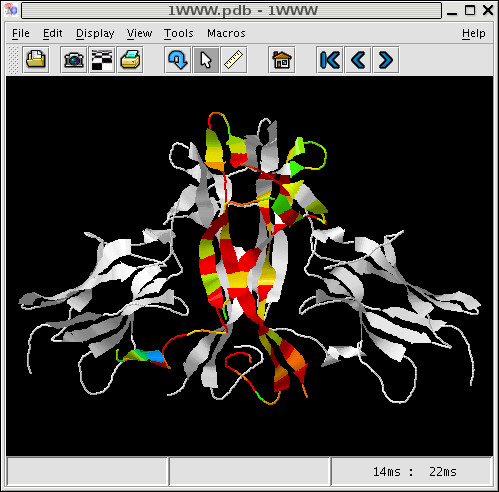
Conservation scores mapped to a structure.

PFAAT uses Jmol [24] to display three-dimensional protein structures. A sequence needs to be associated with PDB file to ensure that the sequence numbering is consistent with the PDB numbering. The PDB sequence is automatically aligned to the PFAAT sequence and the user has the option of fixing any alignment errors. Once the alignment is approved, the PDB numbering is applied to the aligned residues. Secondary structure elements are drawn below the sequence and Jmol is launched. By default, residue selection is synchronized between the alignment viewer and Jmol. This is particularly useful when binding site residues need to be mapped from structure to sequence or vice versa. Once the structure is associated, one can compute residue solvent accessibilities (rsa) [25] which are stored as residue annotations. This feature is useful, if one wants to focus analysis on exposed residues. For example, conservation scores can be computed for a cluster of sequences and mapped to surface residues on the structure (Figure 5) in a similar fashion to the Evolutionary Trace method [26]. A standard color spectrum is applied to the structure, where red is for invariant columns and violet is for the most variable columns.

### Sequence Analysis

There are number of sequence analysis tools that are primarily found under the *Analysis *menu. For example, amino acid percent identities can be computed between all sequences or a subset of their columns. There is also an identity count, which reports the number of sequences that have a residue that is identical to a particular sequence at each column. There are a variety of conservation scores, the default being a von Neumann Entropy based score (described below) that can be applied to selected sequences as well as selected columns. The Conservation scores can be mapped to a 3D structure as discussed above. The PLSR method allows one to identify sequence trends that best correlate with numerical experimental measurements (e.g. binding data that is stored as a sequence annotation). Immediately above each alignment column is a gray box. A single click on a box will show the number and type of residues that are found at a column. In sort mode, the user can select a residue type that will determine how the sequences are sorted. For example, one might be interested in moving all sequences that have a lysine or arginine at column 100 to the top. In filter mode, all sequences that do not have a lysine or arginine would be hidden. The sort mode is often used for mutagenesis experiments as it provides a nice summary of residues that are tolerated at a given position. The filter mode can be used when designing selective drugs for a large gene family. Several other features are described in the documentation on the PFAAT home page.

### Von Neumann Entropy

Although Shannon Entropy is a popular measure of residue conservation, it incorrectly treats amino acids as being orthogonal. Von Neumann Entropy overcomes this shortcoming and is the default measure of residue conservation in PFAAT. Shannon Entropy is described in equation 1, where *i *enumerates each mutually exclusive entity, λ_*i *_> = 0 and Σλ_*i *_= 1. The λ_*i *_are a measure of the probability of encountering the entity *i *in the collection.

Entropy = -Σ*λ*_*i *_log(*λ*_*i*_)

As the 20 amino acids are non-orthogonal (overlapping) vectors, the set must be expressed in terms of an equivalent orthogonal basis set. The mutual overlap of the distinct amino acid vectors in each column is described by a matrix ρ encoding the pairwise similarities between these non-orthogonal vectors. We have found that the following simple 20 × 20 matrix, also called the density matrix, works well for amino acid conservation:

ρ = FS

where F is a diagonal matrix of amino acid 'counts' or frequencies and S is an appropriate amino acid similarity matrix (e.g BLOSUM 62).

Now ρ can be naturally expressed in terms of an orthogonal basis through diagonalization, i.e. by calculating its eigenvectors E and eigenvalues Λ = diag (λ_*i*_) [27]:

ρ = E Λ E^(-1)

The eigenvectors can be interpreted as 20 orthonormal amino acid properties spanning 'amino acid space'. If ρ is normalized such that Trace (ρ) = 1 (i.e. Σλ_*i *_= 1), the eigenvalues λi can be interpreted as the probabilities of encountering each of these 20 orthogonal eigenvector properties in the column. Inserting the eigenvalues λ_*i *_into the formula (1) now gives the entropy measure we desire. The entropy measure can in fact be written directly in terms of ρ itself

Von Neumann Entropy = - Trace (ρ Log ρ)

as can be seen by inserting (3) into (4) to recover (1). Equation (1) is computationally more efficient than equation (4) and is implemented in PFAAT.

## Conclusion

A MSA provides valuable information about a protein family. Additional knowledge is provided by the user in the form of annotations. By combining these annotations with sophisticated analysis, PFAAT allows researchers to test hypothesis that relate to sequence, structure and function. This release of PFAAT marks a significant improvement in functionality over version 1.0. The major improvements are described in the *What's new? *section of the user documentation. We eagerly anticipate user feedback and a 'request features' link is provided on the project home page. Future areas of development might include the extraction of sequence annotations from additional databases (e.g. GO, KEGG, and PFAM) and employing mechanisms to propagate annotations to other sequences [17].

## Availability and Requirements

**Project name: **PFAAT

**Project home page: **

**Documentation: **

**Operating Systems: **Platform independent

**Programming language: **Java 1.4.2 or higher

**License: **GNU General Public License

**Any restrictions to use by non-academics: **None

## Abbreviations

PFAAT – Protein Family Alignment Annotation Tool

MSA – Multiple Sequence Alignment

PLSR – Partial Least Squares Regression

## Competing interests

The author(s) declares that there are no competing interests.

## Authors' contributions

DRC wrote Java code, implemented algorithms, specified and prioritized features, wrote user documentation, and contributed to the writing of the manuscript. VM wrote Java code and re-architected the package. PHD wrote Java code and re-architected the package. MO wrote Java code. SS specified algorithms for implementation and contributed to the writing of the manuscript. YW wrote Java code. EJH wrote Java code and implemented algorithms. SP wrote Java code and implemented algorithms. BEC wrote Java code and implemented algorithms. ESH contributed to the writing of the manuscript. All authors read and approved the final manuscript.
